# THE IMPACT OF DIETARY PROTEIN RESTRICTION ON WHITE ADIPOSE TISSUE FUNCTION DURING AGING

**DOI:** 10.1093/geroni/igae098.3744

**Published:** 2024-12-31

**Authors:** Khristina Young, Molly Easter, Prerana Vaddi, Michael Donkor, Meghan Wong, Cristal Hill

**Affiliations:** University of Southern California, Los Angeles, California, United States; University of Southern California, Los Angeles, California, United States; University of Southern California, Leonard Davis School of Gerontology, Los Angeles, California, United States; Univeristy of Southern California, Los Angeles, California, United States; Univeristy of Southern California, Los Angeles, California, United States; University of Southern California, Los Angeles, California, United States

## Abstract

White adipose tissue (WAT) regulates metabolic health, lipid storage and energy mobilization, yet these functions decline during aging and aging with obesity. Studies show that interventions such as dietary protein restriction (DPR) can improve metabolism, benefit overall health, and extend lifespan. Hence, the unique impact of DPR on the physiological function of adipose tissue represents a plausible target to support general health. Data from our lab and others show that DPR induces browning in WAT through thermoregulatory genes. This modification within the WAT produces beige adipocytes by marked increases in UCP1 (Uncoupling protein 1) gene expression and other related genes, such as the coregulator PR-domain containing 16 (PRDM16). Specifically, PRDM16 regulates adipogenic differentiation, thus playing a key role in regulating the function of adipose tissue. Our goal is to investigate if browning of WAT via Prdm16 is essential to improve metabolic health during DPR, and if DPR-induced improvements in metabolic health are sex dependent. Here, 3-months of age, male and female normal-control littermates and Prdm16 KO mice were fed either a normal protein (20%) or a low-protein (5%) diet for 7 months. Collectively, these data suggest that Prdm16 contributes to dependent and independent metabolic endpoints on body weight, glucose and lipid homeostasis, and adipokine signaling in WAT in middle aged mice. Furthermore, transcriptomic profiling highlights that DPR induces biological processes that are sex-dependent on energy storage and usage. The work is supported by the National Institute on Aging (NIA) of the National Institutes of Health (NIH) - MOSAIC– NIA R00AG070273.

